# Multi-Threshold Recurrence Rate Plot: A Novel Methodology for EEG Analysis in Alzheimer’s Disease and Frontotemporal Dementia

**DOI:** 10.3390/brainsci14060565

**Published:** 2024-06-01

**Authors:** Huang Zheng, Xingliang Xiong, Xuejun Zhang

**Affiliations:** 1 School of Psychological and Cognitive Sciences, Peking University, Beijing 100871, China; 2 Key Laboratory of Child Development and Learning Science, Ministry of Education, School of Biological Science and Medical Engineering, Southeast University, Nanjing 210096, China; xx2404@cumc.columbia.edu; 3 School of Computer, Electronics and Information, Guangxi University, Nanning 530004, China; 4 Guangxi Key Laboratory of Multimedia Communications and Network Technology, Guangxi University, Nanning 530004, China

**Keywords:** multi-threshold recurrence rate plot, recurrence complexity, electroencephalogram (EEG), neurodegenerative disease, support vector machine (SVM)

## Abstract

This study introduces Multi-Threshold Recurrence Rate Plots (MTRRP), a novel methodology for analyzing dynamic patterns in complex systems, such as those influenced by neurodegenerative diseases in brain activity. MTRRP characterizes how recurrence rates evolve with increasing recurrence thresholds. A key innovation of our approach, Recurrence Complexity, captures structural complexity by integrating local randomness and global structural features through the product of Recurrence Rate Gradient and Recurrence Hurst, both derived from MTRRP. We applied this technique to resting-state EEG data from patients diagnosed with Alzheimer’s Disease (AD), Frontotemporal Dementia (FTD), and age-matched healthy controls. The results revealed significantly higher recurrence complexity in the occipital areas of AD and FTD patients, particularly pronounced in the Alpha and Beta frequency bands. Furthermore, EEG features derived from MTRRP were evaluated using a Support Vector Machine with leave-one-out cross-validation, achieving a classification accuracy of 87.7%. These findings not only underscore the utility of MTRRP in detecting distinct neurophysiological patterns associated with neurodegenerative diseases but also highlight its broader applicability in time series analysis, providing a substantial tool for advancing medical diagnostics and research.

## 1. Introduction

Neurodegenerative diseases, such as Alzheimer’s Disease (AD) and Frontotemporal Dementia (FTD), pose complex challenges in neuroscience due to their complex pathologies, which include progressive neuronal loss leading to cognitive decline and altered brain function. AD primarily affects older adults, manifesting as memory loss, confusion, and mood changes, while FTD typically arises in younger adults (45–65 years) and affects personality and language more than memory. These diseases not only differ in symptomatic expression but also in their underlying pathological and neurodynamical mechanisms, including abnormal protein accumulation and neurotransmitter disruption. With an aging global population, the prevalence of AD and FTD is expected to reach 150 million by 2050, presenting significant diagnostic and caregiving challenges [1].

Traditionally, diagnosing Alzheimer’s involves patient history, clinical observations, and cognitive tests like the MMSE [2,3]. Recent research has shifted towards sophisticated imaging techniques such as CT, PET, and MRI [4,5]. However, these methods come with limitations like high costs and potential side effects [6], which restrict their routine clinical application. As an alternative, Electroencephalogram (EEG) analysis has gained prominence, providing non-invasive insights into the brain’s electrical activity and offering potential for early biomarker detection in neurodegenerative diseases. In particular, the nonlinear dynamics of EEG signal analysis have been growing as a hot topic in the study of neurodegenerative diseases, such as AD and FTD [7,8,9]. For instance, EEG complexity measured by permutation entropy was decreased in AD [10]. Alpha band functional connectivity was decreased in patients with AD [11].

Recent advancements in fractal analysis have significantly enriched the study of EEG signals, especially in the context of neurodegenerative diseases [12,13,14]. While the application of the fractal dimension and the Hurst exponent have been applied in the context of neurodegenerative diseases [15,16,17,18,19], these metrics have often been applied in isolation, overlooking their potential synergy. The fractal dimension was interpreted as a measure of structural complexity [20], which quantifies the irregularity, intricacy, and self-similarity of a nonlinear complex system. The Hurst exponent was used to analyze the fractal-like or self-similarity behavior and to quantify long-term memory or persistence in a time series [17,21,22,23], but it sometimes fails to capture the shorter, more transient dynamics prevalent in pathological brain states.

**Figure 1:**
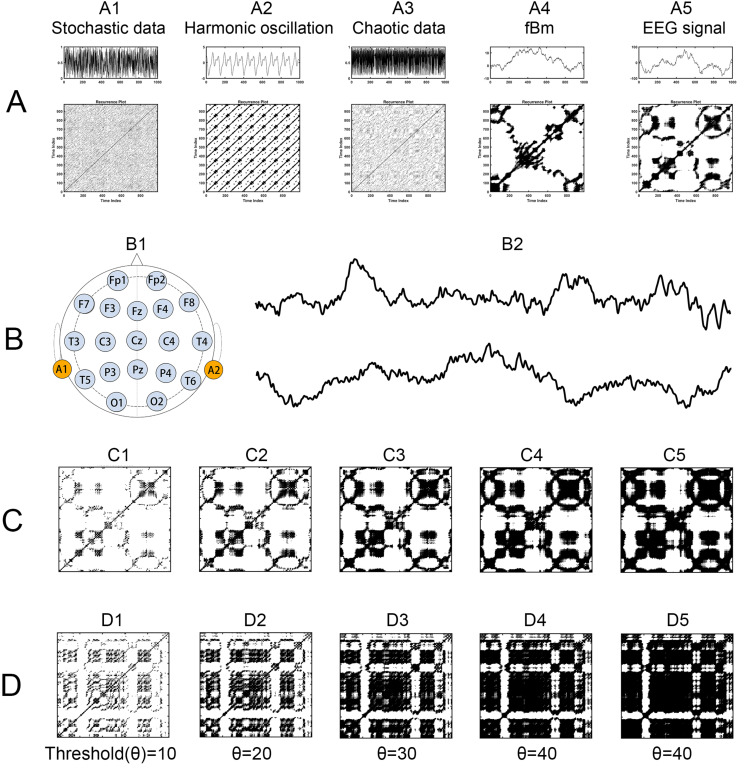
Recurrence Plots for Various Time Series. (**A**) Time series, each followed by its corresponding recurrence plot, including A1 stochastic data, A2 harmonic oscillation, A3 chaotic data, A4 fractional Brownian motion, and A5 EEG signal. (**B**) EEG recording, with B1 showing the distribution of electrodes on the EEG scalp, along with B2 illustrating two EEG time series samples: one from a healthy participant and another from a patient with Alzheimer’s Disease. (**C**,**D**) Recurrence plots with five different thresholds, derived from the EEG time series of a healthy participant and an Alzheimer’s Disease patient, respectively.

**Figure 2:**
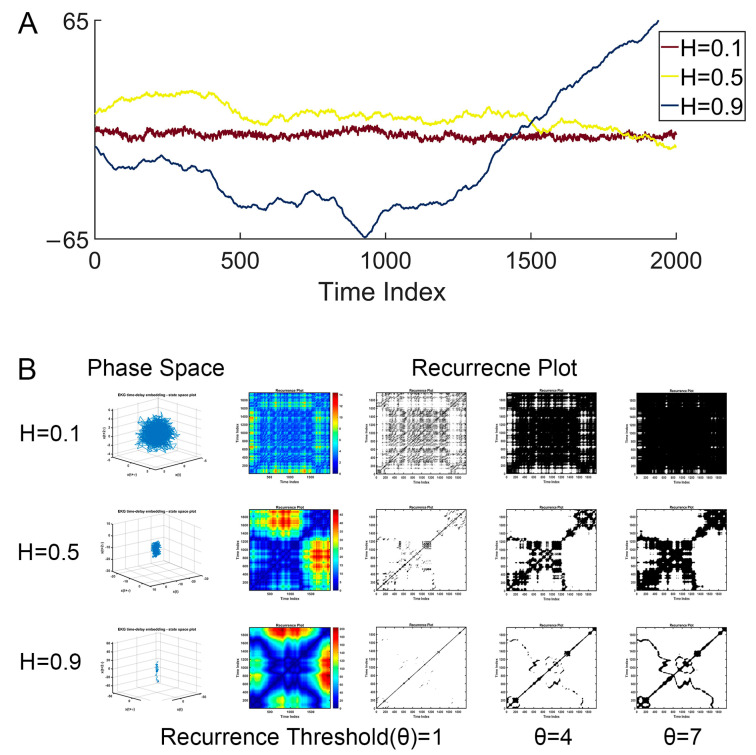
Illustration of fractal Brownian motion and its recurrence plot. (**A**) Examples of fBm with different Hurst exponents. (**B**) The corresponding recurrence plot (under different recurrence thresholds) of the fBM.

Furthermore, neural signals, together with other natural phenomena, often display distinctive recurrent behaviors, ranging from regular to irregular patterns, as illustrated in Figure 1 and Figure 2. Such recurrent behaviors could be characterized by the recurrence plot, a method pioneered by Eckmann et al. [24], but this remains underexplored. The recurrence plot serves as an insightful tool for visualizing the recurring nature of states in a dynamical system’s trajectory through its phase space. Current applications, such as alterations in recurrence structures in Alzheimer’s disease [25] and combined recurrence and cross-recurrence quantification for mild cognitive impairment classification [26], have shown promise but lack a unified approach that encompasses the full spectrum of dynamical behaviors. Recent innovations like the no-threshold recurrence plot convolution network [27] and the multiscale dispersion recurrence plot [28] offer advancements in disease characterization, yet they frequently omit considerations of scale and threshold sensitivity, which are critical for comprehensive analysis.

In addition, multiscale recurrence analysis was proposed and utilized in heart rate variability [29], MEG signals of schizophrenia [30], financial time series [31,32,33], and bridge dynamics [34]. These works focus on the time length (scale) of the input data, while neglecting the threshold of the recurrence quantification analysis. Although the Scale Dependent Lyapunov Exponent (SDLE) developed by Gao and colleagues [35,36] and the correlation integral developed by Grassberger et al. [37] also utilize multiple thresholds of recurrence, they serve distinct purposes within the field of dynamical systems analysis. SDLE was designed to distinguish chaos from noise by characterizing how the Lyapunov exponent changes with increasing recurrence thresholds, and the correlation integral was developed to capture the entropy. In contrast, our Multi-Threshold Recurrence Rate Plot (MTRRP) focuses on capturing the structural complexity and local randomness in EEG signals, particularly in the context of neurodegenerative diseases, offering a different approach and application. This distinct approach not only differentiates MTRRP from existing methods but also highlights its potential utility in medical diagnostics and research into complex neurological conditions. Further, the existing literature lacks a comprehensive exploration of the intersection between recurrence plot analysis, fractal theory, and structural complexity in the context of neurodegenerative diseases.

To bridge this gap, we have created an innovative methodology that integrates fractal analysis with recurrence plot analysis through our novel MTRRP and the Recurrence Complexity metric. This approach is premised on the idea that neurodegenerative diseases induce distinctive alterations in the brain’s electrical activity, which can be elucidated through sophisticated signal processing techniques. MTRRP allows for the dynamic adjustment of recurrence thresholds, revealing changes in recurrence rates that correlate with disease progression. Recurrence Complexity, developed as a product of two novel metrics—Recurrence Rate Gradient and Recurrence Hurst—effectively captures the nuanced interplay between local randomness and global structural features. Recurrence Rate Gradient assesses the initial rate at which similar states become more frequent as the threshold increases. A high Recurrence Rate Gradient suggests a behavior similar to Gaussian noise, thus reflecting local randomness. In contrast, the Recurrence Hurst uses fractal concepts to quantify the long-term memory or persistence of the signal, indicative of underlying global structures.

Our study applies this methodology to resting-state EEG data from patients with Alzheimer’s Disease (AD) and Frontotemporal Dementia (FTD), alongside healthy controls, to identify the distinct neurophysiological patterns that differentiate these conditions. By leveraging the sensitivity of the Recurrence Rate Gradient to subtle changes in recurrence behavior and the depth of analysis provided by the Recurrence Hurst, we enhance the diagnostic capabilities for neurodegenerative diseases, with the generated features being evaluated through a support vector machine (SVM). SVM and other machine-learning approaches have been proved to be promising in the computer-aided diagnosis of degenerative diseases [38,39,40]. This not only provides deeper insights into their neurodynamic processes but also underscores the potential of MTRRP and its metrics to be significant advancements in the field, offering a comprehensive tool for advancing medical diagnostics and research.

## 2. Materials and Methods

### 2.1. Dataset

Resting-state EEG recordings from a dataset of 88 participants (29 AD, 23 FTD, and 36 matched healthy controls) were collected by Miltiadous et al. [41], retrieved from Openneuro Platform (https://openneuro.org/datasets/ds004504), and accessed on 20 June 2023. The demographic statistics can be seen in Table 1, where the Mini-Mental State Examination (MMSE) evaluates cognitive neuropsychological state, which ranges from 0 to 30, with lower MMSE scores indicating more severe cognitive decline. The duration of the disease was measured in months and the median value was 25 with the IQR range (Q1–Q3) being 24–28.5 months. Concerning the AD groups, no dementia-related comorbidities have been reported. Table 1 summarizes the most relevant demographic (i.e., age and gender) and clinical (i.e., MMSE score) features of the three groups.

### 2.2. EEG Data Acquisition

Resting-state EEG (rsEEG) data were acquired from the 2nd Department of Neurology of AHEPA General Hospital of Thessaloniki by an experienced team of neurologists, using a Nihon Kohden EEG 2100 clinical device equipped with 19 scalp electrodes (Fp1, Fp2, Fz, F3, F4, F7, F8, Cz, C3, C4, T3, T4, T5, T6, Pz, P3, P4, O1, O2) following the 10–20 international system (see Figure 1B). Two reference electrodes (A1 and A2) were positioned on the mastoids for impedance verification. Prior to each recording, skin impedance was maintained below 5 kΩ. The EEG signals were sampled at 500 Hz with a 10 μV/mm resolution. Recording montages included anteroposterior bipolar and referential configurations, utilizing Cz as the common reference. This experiment and the data collection were approved by the Scientific and Ethics Committee of AHEPA University Hospital, Aristotle University of Thessaloniki, with the protocol number 142/12-04-2023. For analysis, a 5 min segment of data was selected for each participant, as the shortest recording duration was 5.1 min.

### 2.3. EEG Data Preprocessing

The EEG signal preprocessing encompassed several steps. Initially, a Butterworth bandpass filter (0.5 to 45 Hz) was applied, and the signals were referenced to the mean of channels A1 and A2. Subsequently, EEGLab [42], a open source MATLAB (version 2023a) tool for analysis of single-trial EEG dynamics including independent component analysis and other elements of EEG data preprocessing, was employed for artifact correction using the artifact subspace reconstruction approach. This step entailed the removal of data segments exceeding the threshold of standard deviation within a 0.5 s window (capped at 17). Independent component analysis (ICA) was performed using the RunICA algorithm, resulting in the transformation of the 19 EEG signals into 19 distinct independent components. It is noteworthy that despite the recordings being acquired during subjects’ rest with closed eyes, some signals exhibited evidence of eye and jaw movements. Consequently, components identified by EEGLab’s ICLabel routine as associated with eye or jaw movements were automatically excluded from the analysis.

### 2.4. Recurrence Plot

Recurrence plot [24] is a two-dimensional representation characterizing the dynamic features of nonlinear systems and complex time series, by which the phase space trajectory returns roughly to its previous states. For each moment *x*(*i*), estimate the distance of every other time point, see *x*(*j*), and plot the points (*i*, *j*) on the recurrence plot as a revisit if the distance is short enough (shorter than a threshold). This can be processed by the binary recurrence matrix:

(1) $${R\left(i,j\right)=}_{0\;\;\;\;otherwise}^{1\;\;\;\;if\;\left|\left|x\left(i\right)-x\left(j\right)\right|\right|\leq \varepsilon }$$

In this matrix, ||·|| represents a norm, and *ε* is the predefined recurrence threshold. The recurrence plot visualizes this matrix (see Figure 1), typically using colored dots at coordinates (*i*, *j*) to indicate a recurrence (*R*(*i*,*j*) = 1), with time on both the *x*- and *y*-axes.

The detailed calculation is described as follows:

(1) For a given time series, *x*1, *x*2, *x*3,…, *x* = *n*, reconstruct its phase space vectors *X* using time-delay methods, such as taken time delay, in which parameters m and τ for phase space reconstruction should be set. Immediately, we obtain phase space vectors *Xi* = (*xi*, *xi* + *τ*,…, *xi* + (*m* − 1) × *τ*).
(2) Define the parameters of phase space reconstruction m and *τ* (as described in the first step above) and distance threshold *r* for judging whether a points-pair is close enough to take as a recurrence.
(3) Calculate the distance of all point-pairs and generate an *n* × *n* distance matrix, and optionally visualize the matrix.
(4) Plot all of the points-pairs closer than the threshold *r*, and we immediately obtain the so-called recurrence plot, shown in Figure 1.
(5) Estimate various nonlinear dynamic features of the recurrence plot using recurrence quantification analysis (RQA) statistics [24], such as recurrence rate (RR), determinism (DET), entropy (ENTR), MaxLine, Trend, Laminarity, Trapping Time. Recurrence rate quantifies the density of recurrence points in a recurrence plot, that is, the rate of recurrence points divided by all points in the recurrence plot. This rate corresponds to the likelihood of a specific state reoccurring in the system and is closely related to the correlation sum concept.

### 2.5. Fractional Brownian Motion and Hurst Exponent

**Figure 3:**
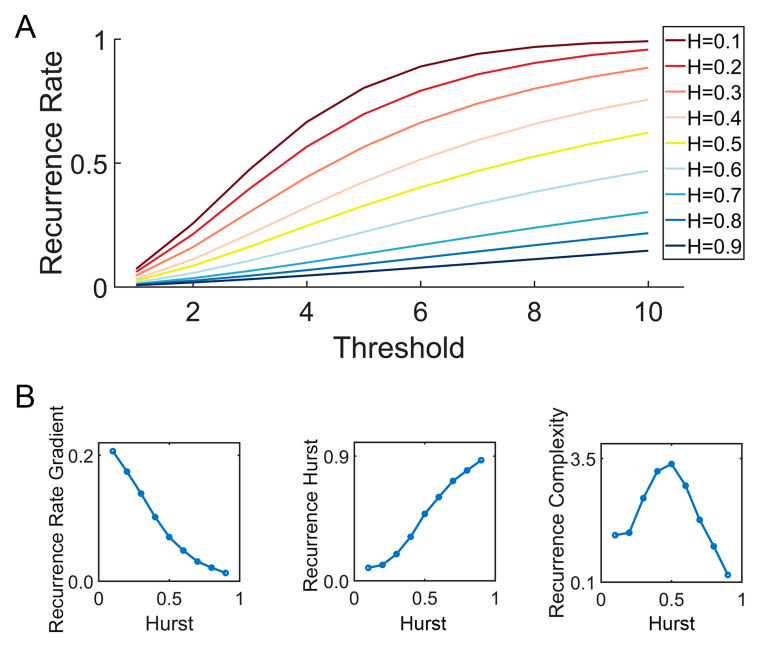
Illustration of the Multi-Threshold Recurrence Rate Plot (MTRRP) and metrics based on MTRRP. (**A**) MTRRP of fBm with different Hurst exponents ranging from *H* = 0.1 to *H* = 0.9. (**B**) How the Hurst exponent of fBm correlates with the Recurrence Rate Gradient, Recurrence Hurst, and Recurrence Complexity.

Fractional Brownian motion (fBm) is a continuous-time Gaussian process that is a cornerstone in the field of stochastic processes, particularly useful in modeling scenarios with self-similarity and long-range dependence. In this study, fBm signals with different levels of long-term dependence were generated by wfbm, a wavelet-based fBm procedure, by setting the Hurst exponent as input parameter. As illustrated in Figure 2, For different fractional Brownian motions (characterized by varying Hurst exponents), the recurrence plot varies as the recurrence threshold is altered. Especially, Hurst exponent decides how the recurrence rate (a simple recurrence quantification metric) evolve with increasing recurrence thresholds (see Figure 3).

The Hurst exponent (*H*) is a measure of fractal complexity and the long-range persistence of the fractal process. There are 3 basic dimensions of Hurst: (1) 0 < *H* < 0.5, an EEG time series exhibiting anti-persistent correlation, or the tendency to recover to the mean value; (2) *H* = 0.5, indicating that an EEG time series is memoryless or a random walk process; (3) 0.5 < *H* < 1, a time series that is considered to have long-term correlation. There are several methods to measure *H*, such as Detrended Fluctuation Analysis [43] and Adaptive Fractal Analysis (AFA) [21], and the AFA is applied in this study. AFA uses an adaptive detrending algorithm to measure the fit between the algorithm-generating trend and the real data at different detrending windows. The detailed calculation is the following:

$$\sum_{j=1}^{i}x(j)$$

(1) For a given time series, *x*(1), *x*(2), *x*(3),…, *x*(*n*), integrate it from a fractional Gaussian motion into a fractional Brownian motion by *X*(*i*) = $\sum_{j=1}^{i}x(j)$
(2) Fit the time series *X*(*i*) using the weighted time window *W* = 2*n* + 1, with the highest weight in the center point of the window, and the weights decaying linearly towards both left and right sides. Thus, we obtain the fitting curves under each fitting window.
(3) Linearly fit the Log2(*W*) and Log2(*F*(*W*)), where *F*(*W*) is the variance of the magnitude of the residuals according to the following:

(2) $$F(W)=[\frac{1}{N}\sum_{i=1}^{N}{\left(u(i)-v(i)\right)}^{2}{]}^{1/2}\;\;\;\sim \;\;\;W^{H}$$

where the power coefficient *H* is the Hurst exponent, which is the slope of the linear fit between Log2(*W*) and Log2(*F*(*W*)).

### 2.6. MTRRP and Recurrence Complexity

In this section, we introduce MTRRP and the derived metrics, including the Recurrence Rate Gradient, Recurrence Hurst, and Recurrence Complexity.

#### 2.6.1. Methodology of MTRRP Construction

**Figure 4:**
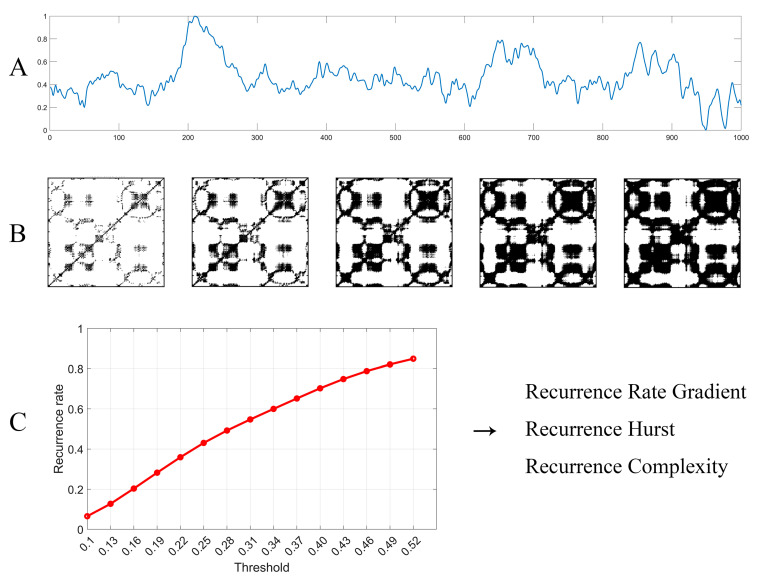
Flow diagram of MTRRP and its derived metrics. (**A**) EEG time series, which was normalized to ranges from 0 to 1. (**B**) The recurrence plot of the time series with six different thresholds. (**C**) The MTRRP, where each point stands for the recurrence rate under a specific threshold, where the recurrence rate can be obtained through the recurrence plot, as illustrated in (**B**) above. Finally, metrics based on MTRRP can be obtained immediately, including the Recurrence Rate Gradient, Recurrence Hurst, and Recurrence Complexity.

As the flow diagram of MTRRP illustrated in Figure 4A shows, the construction of the MTRRP begins with the preparation of the time series data, as depicted in Figure 4. In order to setting appropriate thresholds, the original time series data can be normalized to ranges 0 to 1 by the following formula:

(3) $$normalized\;data=\frac{origninal\;data-minimum}{maximum-minimum}$$

**Figure 5:**
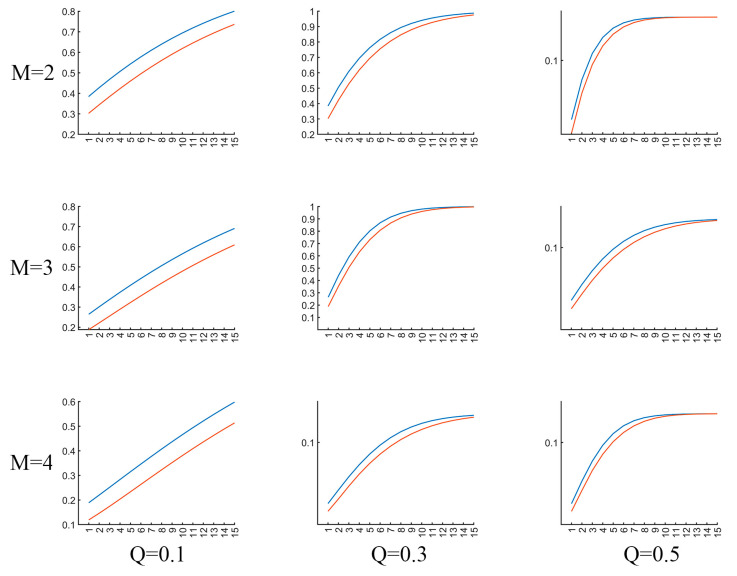
Illustration of MTRRP with different parameters. The blue line and red line are MTRRPs for a piece of EEG signals from AD and healty controls, repsectively. *M* refers to the embedding dimension for phase space construction, *Q* the factor proportional to the standard deviation of the dataset.

It is important to appropriately set the parameters for MTRRP, including the embedding dimension and delay time for constructing a phase space, and the threshold for the recurrence plot. As illustrated in Figure 5, the parameters influence the MTRRP significantly, with the recommended embedding dimension (*M*) being 2 or 3 and the recommended factor (*Q*) of the threshold being 0.3. The embedding dimension, M, is the number of delayed versions of the time series used to reconstruct the phase space. It should be large enough to unfold the dynamics of the system but not so large as to complicate the model unnecessarily. Methods to select M include False Nearest Neighbors. This method involves incrementally increasing mm and checking whether points that are close in mm-dimensional space remain close when the dimension is increased to *m* + 1. The smallest mm for which a small percentage of points are false neighbors is chosen as the embedding dimension. The delay time, *τ*, determines how much the time series is shifted to construct the vectors in the reconstructed phase space. A well-chosen *τ* ensures that the vectors provide meaningful and independent information about the dynamics of the system. Two common methods to determine τ are Autocorrelation Function and Mutual Information. Like the parameters for phase space, choosing appropriate thresholds for MTRRP is crucial. Intuitively, thresholds should be set according to the statistical properties of the data, such as the range and standard deviation.

The initial threshold, defined as a minimum threshold, is set at a predetermined percentage of the total data range, typically 10% (or just 0.1, if the data have been normalized to ranges from 0 to 1). Subsequent thresholds are determined by incrementally increasing this base threshold by a factor (*Q*) proportional to the standard deviation of the dataset, thus ensuring sensitivity to the inherent variability of the data. Each threshold defines a specific condition under which the recurrence of the time series is analyzed using recurrence plot techniques or recurrence quantification analysis [24]. The recurrence rates obtained at each threshold are then plotted against their respective thresholds, forming the MTRRP, which visually represents how the recurrence behavior of the data evolves with increasing thresholds, as illustrated in Figure 4C.

The formula used to characterize the MTRRP, based on the simulated data of various fBms, is given by the following:

(4) $$Recurrence\;Rate=\alpha \times \left(1-Recurrence\;Hurst\right)\times \log(threshold)$$

where α is a user-defined constant (typically 0.5 for classical fWm), and the Recurrence Hurst, a measure directly estimating the Hurst exponent from the data, is computed as follows:

(5) $$Recurrence\;Hurst=1-\frac{Recurrence\;Rate}{\alpha \times \log(Recurrence\;Threshold)}$$

#### 2.6.2. Calculation of Recurrence Rate Gradient

The Recurrence Rate Gradient is quantified through linear regression analysis of the initial segment of the MTRRP. This segment’s slope provides a measure of how quickly the recurrence rate increases with the threshold increments and is critical for identifying local randomness in the time series data. The choice of the largest threshold for the fitting process is pivotal and can be determined in two principal ways:

1. By setting a uniform maximum threshold across the dataset that allows most data points to reach a recurrence rate plateau.
2. By customizing the maximum threshold for each specific time series based on the stabilization point of its recurrence rate.

#### 2.6.3. Recurrence Hurst Calculation

Additionally, we compute the Recurrence Hurst—a measure that estimates the Hurst exponent directly from the time series data. As depicted in Formulas (4) and (5), this computation is integrated into the MTRRP analysis to assess the long-term memory or persistence characteristics of the time series, thereby complementing the Recurrence Rate Gradient.

#### 2.6.4. Definition of Recurrence Complexity

Finally, Recurrence Complexity is defined as the product of the Recurrence Rate Gradient and Recurrence Hurst. This product captures both the local randomness and the persistence behavior, reflecting the overall structural complexity of the time series. Higher values of the Recurrence Rate Gradient indicate greater local randomness, whereas higher Recurrence Hurst values suggest pronounced long-term memory or persistence in the data.

These methodological steps and calculations are crucial for providing a comprehensive and nuanced understanding of the dynamical properties of EEG signals, particularly in the context of neurodegenerative diseases. This approach not only enhances traditional EEG analysis but also leverages advanced metrics to offer deeper insights into the underlying complexities of neurological conditions.

## 3. Results

### 3.1. ANOVA Analysis

**Figure 6:**
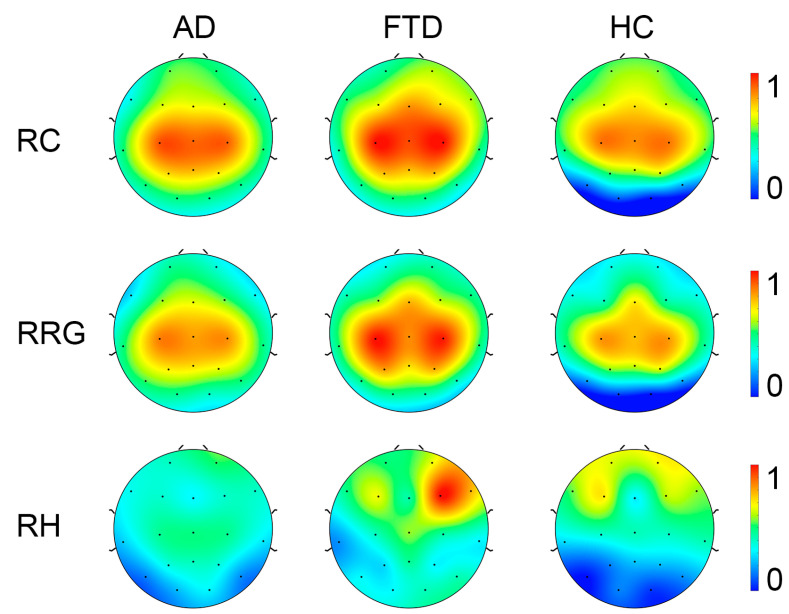
Topography of Recurrence Complexity (RC), Recurrence Rate Gradient (RRG), Recurrence Hurst (RH) in AD, FTD, and healthy controls (HC) with full-band EEG data. For visualization, the metrics were normalized to a range from 0 to 1.

For full-band EEG data (see Figure 6), ANOVA analysis (Bonfferoni corrected for all multiple comparison issues in this paper) of the recurrence complexity revealed a significant group difference in the occipital region (F(2.85) = 6.249, *p* = 0.003, η^2^p = 0.128, 90% CI = [0.029, 0.229]). To determine the specific differences between the three groups, pairwise comparisons were conducted. Both patients with Alzheimer’s Disease (AD) and Frontotemporal Dementia (FTD) exhibited significantly higher complexity than healthy controls (*p* = 0.006 and *p* = 0.014, respectively). Similarly, for the Recurrence Rate Gradient, a significant group difference was detected in the occipital region (F(2.85) = 3.691, *p* = 0.029, η^2^p = 0.080, 90% CI = [0.005, 0.170]). Pairwise comparisons only reached marginal significance between AD and FTD (*p* = 0.050 and *p* = 0.082, respectively). However, the statistical analysis of the Recurrence Hurst provided no evidence of significant group differences (ps > 0.447).

**Figure 7:**
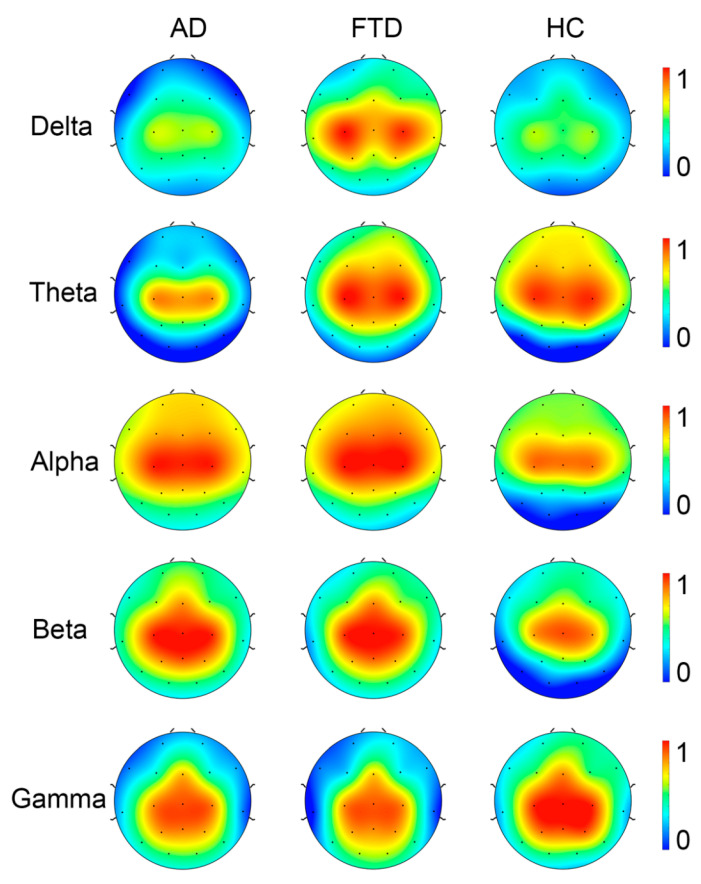
Topography of Recurrence Complexity (RC) in AD, FTD, and healthy controls (HC) at Delta, Theta, Alpha, Beta, and Gamma bands.

**Figure 8:**
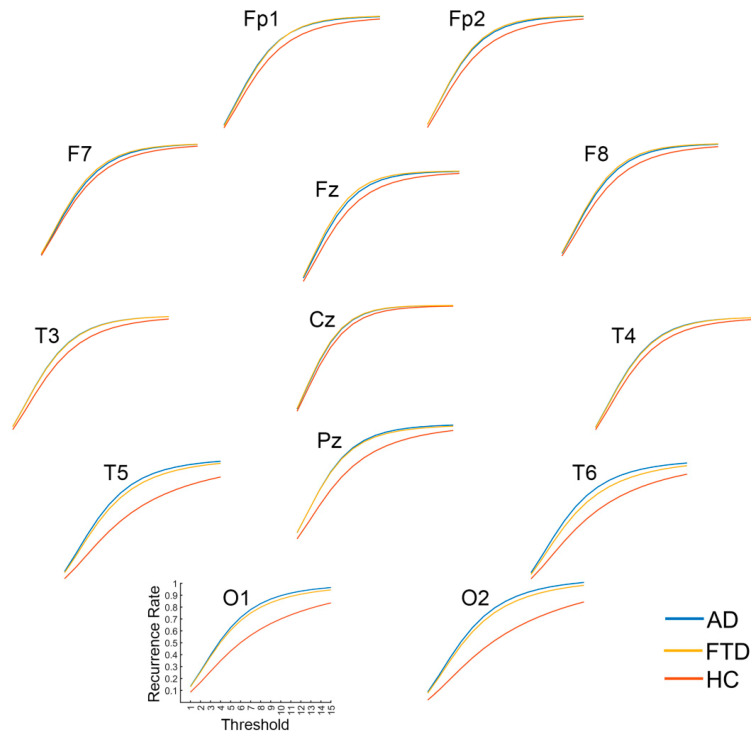
Multi-Threshold Recurrence Rate Plots for Alpha band signals in AD, FTD, and HC.

In consideration that the recurrence complexity is the most effective indicator for the group differences of full-band data, we focus on the group differences in recurrence complexity on band-passed data. As shown in Figure 7, in comparison with healthy controls, both the AD and FTD groups showed significantly increased recurrence complexity on the occipital lobe at the Alpha band (*p* < 0.001 and *p* = 0.011) and the Beta band (*p* < 0.001 and *p* = 0.001), while there was no significant group difference in occipital recurrence complexity in the Delta, Theta, and Gamma bands. Considering the group difference at the alpha band are the most significant, we present the MTRRP of alpha band signals from AD and healthy control (see Figure 8).

### 3.2. SVM Classification

To test the effectiveness of our proposed method in diagnosing AD and FTD, SVM was used for classification tasks, using the recurrence complexity, Recurrence Hurst, and Recurrence Rate Gradient as input features. By applying leave-one-out cross-validation, we observed a promising classification performance, as seen in Table 2. The strategy for feature selection involved calculating Pearson’s correlation between the features and group labels. Features demonstrating a stronger correlation were considered to have a potentially higher predictive value for classification. We tested the top 50 features (from 285 features = 19 EEG channels × 5 frequency bands × 3 metrics, for each subject) with the highest correlation in a SVM, and the classifier attained an accuracy of 86.36% in distinguishing AD and FTD from HC, an accuracy of 87.69% in distinguishing AD from HC, an accuracy of 82.69% in distinguishing FTD from HC, and an accuracy of 72.88% in distinguishing AD from FTD. The details of the classification performance are listed in Table 2, including accuracy, sensitivity, and specificity.

**Figure 9:**
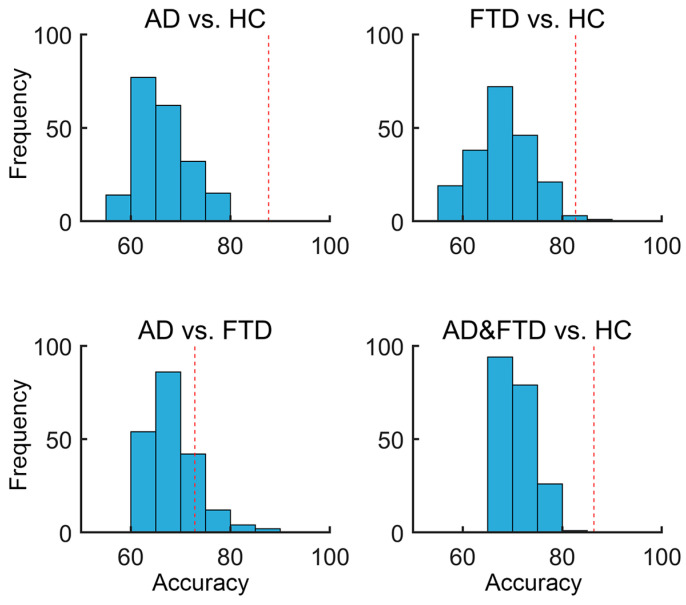
Null distributions for the SVM permutation test by shuffling group labels. The *X*-axis represents the classification accuracy, while the *Y*-axis shows the frequency, indicating the number of occurrences within each accuracy bin. The red dashed vertical line represents the classification accuracy with the real group labels of the training data.

To access the statistical significance of the classification results, a permutation test was performed by randomly shuffling the group labels of the samples. Specifically, each time, we shuffled the labels and trained a new SVM classification model based on the new data, thus obtaining and recording the performance of the classifier. The strategies and specifics of training–testing in the permutation test section were performed identically to the experiment using the real group labels, as reported above (e.g., in each shuffling, features were ranked by correlation coefficient with the shuffled labels, and the top 50 correlated features were selected as input features). This process was repeated 200 times to obtain a null distribution for each classification task. As presented in Figure 9, the permutation test result showed a significantly lower classifier performance in HC vs. AD (*p* < 0.001), HC vs. FTD (*p* = 0.015), and HC vs. AD and FTD (*p* < 0.001), but not in AD vs. FTD (*p* = 0.19), compared to the classification performance using real labels (see Figure 3 and Table 2).

## 4. Discussion

Our study introduces a novel methodology, termed the Multi-Threshold Recurrence Rate Plot (MTRRP), as well as the generated metrics, including Recurrence Complexity, Recurrence Rate Gradient, and Recurrence Hurst. This is a new approach for EEG signal analysis in Alzheimer’s Disease (AD) and Frontotemporal Dementia (FTD), bridging recurrence quantification analysis [24] with fractal theory [23]. By bridging recurrence plot analysis with fractal theory, this approach offers a nuanced understanding of EEG patterns, significantly enhancing the diagnostic accuracy for neurodegenerative conditions. Specifically, the application of Recurrence Complexity to resting-state EEG data highlighted how the pronounced complexity increases in the occipital regions of patients with AD and FTD, particularly in the Alpha and Beta frequency bands. Further, the efficacy of our methodology was tested in SVM, which demonstrated high classification accuracies of 87.7% for AD and 82.7% for FTD, underscoring the potential of MTRRP in medical diagnostics.

By bridging fractal analysis and recurrence plot analysis in EEG signals, the diagnosis of Alzheimer’s Disease (AD) is achieved by integrating the methodologies of both fields. Fractal analysis, with its focus on understanding complex patterns at various scales, complements the recurrence plot analysis, which visualizes the recurrence of states in a dynamical system. The two samples of the EEG time series shown in Figure 1B, which look similar but vary considerably in MTRRP, are depicted in Figure 1C,D. The use of multiple thresholds in our MTRRP reflects the ideas of multiscale analysis inherent in fractal theory. This allows for a more nuanced examination of EEG signals, capturing variations across different scales, which is critical in understanding the complex dynamics of neurodegenerative diseases. This synergy between fractal analysis and recurrence plot analysis offers a comprehensive tool for examining the intricate patterns present in the EEG data of AD patients, potentially leading to more accurate diagnoses and insights into the disease’s progression.

The observation of increased Recurrence Complexity in both AD and FTD within the occipital regions is a significant finding. This suggests a distinctive alteration in the dynamical behavior of the EEG signals in these areas, which are typically associated with visual processing. Previous studies have employed various entropy and complexity measures in EEG data, such as Lempel–Ziv complexity [7], correlation dimension [37], and maximum Lyapunov exponent [15]. For instance, the Lempel–Ziv complexity [7] and entropy [44,45] were significantly decreased in AD at the parietal and occipital regions, and these complexity changes can aid in the diagnosing of AD with 90% sensitivity and 73% specificity [7]. Morison et al. [46] demonstrated that permutation entropy, a simple robustness measure widely used in quantifying observations and dynamic noise, is a good biomarker for differentiating AD patients from healthy controls. Smits et al. [16] reported that AD exhibited a decreased fractional dimension in the temporal–occipital regions at resting state. However, these studies did not specifically focus on differentiating long-term memory as an index by Hurst exponent values or randomness-based complexity among AD, FTD, and healthy controls. Our study addresses this gap by exploring the nonlinear dynamics in neural oscillations of AD and FTD, contributing to a deeper understanding of the mechanisms underlying these neural disorders.

In this study, the application of MTRRP for the SVM classification of EEG signals in neurodegenerative diseases achieved a classification accuracy of 87.7%. While this may not reach the near-perfect accuracies reported in other conditions such as epilepsy detection [40,41], it is important to contextualize these results within the broader landscape of EEG analysis in neurodegenerative disorders, as the epilepsy signal is very different to the normal controls and can be much more easily detected. Recent advancements in machine-learning approaches for EEG analysis have shown promising results. For instance, Prado et al. [47] reported impressive classification performances using multi-metric rsEEG source–space connectivity features. However, their study was limited by using a single data split for training and testing, which might not adequately reflect the robustness of the classifier across different subsets of data. On the other hand, Vecchio et al. [38] achieved a notable accuracy of 95% in distinguishing Alzheimer’s Disease (AD) from healthy controls using EEG connectivity metrics. However, their approach of using data segments rather than whole-subject levels might risk data leakage and affect the generalizability of their findings.

The implications of this result are twofold. Firstly, it affirms the potential of Recurrence Complexity as a sensitive biomarker for neurodegenerative diseases. Secondly, it suggests that AD and FTD may involve more widespread neural network disruptions than previously thought, extending beyond the traditionally affected regions. This finding could lead to a deeper understanding of the pathophysiological mechanisms underpinning the neurodegenerative processes of these diseases and might influence future approaches to diagnosis and treatment. However, our study does face certain limitations. The sample size and diversity may affect the generalizability of our findings. Further research with a larger and more varied cohort is essential to validate our results. Additionally, integrating our approach with other diagnostic tools, such as MRI and PET scans, could offer a more comprehensive diagnostic framework. Future research should also explore the application of Recurrence Complexity in other neurological conditions, potentially offering a universal tool for neurophysiological analysis. Longitudinal studies could provide insights into the progression of neurodegenerative diseases and the efficacy of treatment interventions.

The versatility of the MTRRP extends well beyond the scope of EEG signal analysis in neurodegenerative disorders, opening new avenues across a broad spectrum of time series analysis. The methodology is equally applicable to financial time series analysis, such as monitoring stock market trends, as well as to evaluating bridge dynamic properties in civil engineering and analyzing heart rate variability in cardiology. This adaptability underscores the potential of MTRRP to revolutionize the way dynamic systems are analyzed across various disciplines by providing a detailed and nuanced understanding of underlying patterns. Moreover, the current study has focused primarily on the recurrence rate within the framework of multi-threshold recurrence plots. Future investigations could expand this approach by incorporating other metrics of recurrence quantification analysis such as Determinism, Maximum Line Length, Entropy, Trend, Laminarity, and Trapping Time. Exploring these metrics could further enhance the diagnostic capabilities of MTRRP, offering a more comprehensive toolset for analyzing complex time series data across different fields. This expansion not only broadens the applicability of MTRRP but also enhances its theoretical foundation, paving the way for its adoption in diverse scientific and practical applications.

## 5. Conclusions

In conclusion, our study proposes the Multi-Threshold Recurrence Rate Plot (MTRRP), and we applied this innovative methodology for EEG analysis in Alzheimer’s Disease (AD) and Frontotemporal Dementia (FTD). Utilizing metrics like Recurrence Complexity, which integrates Recurrence Rate Gradient and Recurrence Hurst, this approach highlights significant alterations in the EEG dynamics of the occipital regions in AD and FTD, particularly within the Alpha and Beta frequency bands. The application of MTRRP resulted in a classification accuracy of 87.7%, demonstrating its potential as a diagnostic tool. This methodology not only advances EEG analysis by bridging recurrence plot and fractal analysis but also opens new avenues for understanding the complex dynamics of neurodegenerative diseases. Future research should expand the sample size and integrate MTRRP with other diagnostic methods to enhance its effectiveness and applicability in clinical settings.

## Figures and Tables

**Table 1:** Demographic and clinical data of the healthy control (HC), Alzheimer’s disease (AD), and frontotemporal dementia (FTD) participants.

Group	Gender (M/F)	Age	MMSE
AD	12/24	66.39 ± 7.89	17.75 ± 4.50
FTD	9/14	63.65 ± 8.22	22.17 ± 2.64
HC	11/18	67.90 ± 5.40	30.00 ± 0.00

**Table 2:** Classification performance using SVM.

	Accuracy (%)	Recall (%)	Specificity (%)	Null Distribution Accuracy (Mean ± sd)
HC/AD	87.69	97.22	75.86	66.50 ± 5.46
HC/FTD	82.69	73.91	89.66	68.01 ± 5.48
AD/FTD	72.88	94.44	39.13	68.53 ± 5.10
HC/AD&FTD	86.36	93.22	72.41	70.78 ± 3.11

## Data Availability

Datasets used in this study are openly available at https://openneuro.org/datasets/ds004504 (accessed on 20 June 2023).
